# User Perceptions of E-Cigarette Cessation Apps: Content Analysis of App Reviews

**DOI:** 10.2196/59997

**Published:** 2025-04-15

**Authors:** Danielle Rodberg, Roula Nawara, Mischa Taylor, Laura Struik

**Affiliations:** 1 School of Nursing University of British Columbia Kelowna, BC Canada

**Keywords:** qualitative research, vaping, e-cigarette, mobile phone, mHealth, smartphone app, cessation, nicotine, consumer, perception, user, content analysis, Canada, experiences

## Abstract

**Background:**

Vaping rates in Canada are continuing to increase. In 2019, 4.7% of Canadians used an electronic cigarette (e-cigarette) in the past 30 days, which rose to 5.8% in 2022. In the same year, young adults aged 20-24 years demonstrated the highest use among Canadians, at 19.7%. Given this, existing interventions are not resulting in the desired outcomes, and smartphone apps have the potential to address this gap. Although limited, current evidence highlights that apps can be an effective cessation support; however, a gap persists in understanding the user experience of vaping cessation apps.

**Objective:**

The purpose of this study was to explore the user experience of vaping cessation apps through an analysis of app reviews. More specifically, this study aimed to identify positive and negative experiences of app users, as well as highlight recommendations from app users to improve the quality of these apps.

**Methods:**

Vaping cessation apps were identified through searches on the Canadian and US versions of Apple App Store and Android Google Play Store in August 2022. Searches revealed a total of 11 vaping cessation apps with app reviews, which resulted in a total of 310 reviews for analysis. Review material was analyzed using a deductive content analysis approach and divided into the following primary categories: content, functionality, aesthetic, cost, and other. These were further divided into 3 secondary categories (praise, criticism, and recommendations) and various tertiary categories.

**Results:**

The most discussed primary categories were content, functionality and cost. Comments regarding content tended to be positive (n=103, 33.2%), praising features, such as hypnosis audio sessions (n=29, 28.2%) and tracking features. In contrast, comments tended to criticize functionality (n=58, 18.7%), indicating issues with the functioning of an app that either made the whole app unusable (n=29, 50%) or a specific feature unusable (n=28, 48.3%). Reviews regarding cost were mixed, with 27 (8.7%) positive comments, the majority of these encompassing reviewers satisfied with their purchase (n=17, 63%), and 38 (12.3%) negative comments, including individuals both unsatisfied with their purchase (n=15, 39.5%) and unsatisfied with the free version (n=12, 31.6%).

**Conclusions:**

This study is the first of its kind to evaluate the user experience with vaping cessation apps via an analysis of app reviews. App developers may benefit from reading our findings to identify areas to focus on when developing and updating apps. Our study forms a basis for the development of future vaping interventions, as well as future studies. Future research should be conducted on vaping cessation interventions with an emphasis on the user experience because there is limited research available for comparison with the promising results from this study.

## Introduction

Vaping rates continue to rise in Canada despite efforts to prevent vaping uptake and support cessation efforts [1]. In 2022, 5.8% of the population reported using an electronic cigarette (e-cigarette) in the past 30 days, up from 4.7% in 2019 [1,2]. Vaping rates among youth and young adults are disproportionately higher compared to the adult population, with 13.6% of youth aged 15-19 years and 19.7% of young adults aged 20-24 years vaping in the past 30 days compared to 3.9% of Canadians aged 25 years and older [1]. Despite these high rates, this population demonstrates great interest in cessation supports, with over 100,000 youth and young adults registering for a text messaging support intervention (“This is Quitting”) a year after it was introduced to the public [3]. Although limited, emerging evidence indicates that smartphone apps are a promising avenue to deliver vaping cessation support [4-9].

The use of smartphones continues to increase worldwide. According to a recent study, 85% of individuals from 19 economically advanced countries in Europe, North America, and Asia use a smartphone [10]. Among Canadians specifically, this rate is 84% [10]. Unsurprisingly, rates of smartphone usage are high among younger populations, with 98% of Canadians aged 18-29 years owning a smartphone [10]. The demand for mobile health (mHealth) apps is continuously increasing, with app usage spiking since the COVID-19 pandemic [11], and cessation apps are no exception. For instance, English-language smoking cessation apps have been downloaded over 33 million times since 2012 [12]. However, it is important to note that to the best of the authors’ knowledge, there are no data available on the number of downloads of vaping cessation apps specifically.

Health apps allow individuals to be active participants in their health and provide them with the resources needed to induce change in their behaviors [6]. Studies show that participants report the use of apps to be an overall positive experience and find them easy to use, and apps increase the equity and accessibility of interventions, with many participants preferring apps over traditional behavior change interventions (eg, face-to-face counseling) [4,6,7]. Indeed, there are a variety of benefits to using apps over traditional interventions, including reduced barriers to access, which enables apps to reach a wider audience [8,13-15]. When compared with in-person support interventions, apps are more cost-effective and can be used at one’s convenience whenever needed [8,15]. In addition, apps can be developed in a manner that enables the user to customize their experience [8]. Nevertheless, even though evidence shows apps have the potential to increase behavior change, many smartphone apps are only used once after being installed [14]. This highlights the importance of understanding what mechanisms and features are contributing to positive or negative experiences and ultimately contributing to app retention [14].

Although there are limited studies exploring the efficacy of various behavioral interventions as vaping cessation strategies, there are even fewer studies evaluating apps for their support in vaping cessation. However, from the limited literature available, studies have revealed promising results [15-17]. For example, a recent study on a text messaging intervention, which provided users with quitting strategies, found that abstinence rates of vaping increase by 6% with the use of this intervention [17]. Other studies have evaluated vaping cessation apps for their use of evidence-based guidelines [8,14]. Further research is needed to determine whether smartphone-based interventions result in higher rates of abstinence from vaping than other behavioral interventions. Furthermore, a persistent gap in the vaping cessation literature is the user experience, which is critical to understanding why some apps succeed and others fail. One aspect of apps that is often overlooked in cessation research are user reviews, which hold valuable information about user experiences [18,19]. Studying reviews helps to understand the target audience of these apps and what features of the apps contribute to a positive or negative experience [18]. This study aimed to explore the user experience of cessation apps that address vaping through an analysis of user reviews. Specifically, this paper identified positive and negative user experiences, as well as recommendations from users to improve the quality of these apps.

## Methods

### Ethical Considerations

No personal information about reviewers was included in this study. As such, this study was classified by the University of British Columbia Okanagan’s Behavioral Research Ethics Board as research not involving human subjects and was, therefore, not subject to institutional review board jurisdiction [20].

### Data Collection

Similar to other studies involving app reviews [21], the app reviews examined in this study are publicly available in Apple App Store and Android Google Play Store. Searches were conducted on both the Canadian and US versions of Apple App Store and Android Google Play Store to gather a list of vaping cessation support apps. The following search terms were used: “quit vaping,” “vaping,” “vaping cessation,” “e-cigarette,” and “quit e-cigarette.” The search strategy intended to mirror the process of app store optimization, which determines how apps are ranked and discovered when customers conduct searches [22]. These search terms were based on the recommendations as per the Apple developer support page to use words a customer would use and included the category or type of app and the use of the app [22]. Apple App Store and Android Google Play Store differ slightly in their optimization methods. Apps are discoverable on Apple App Store based on what keywords developers choose to use in the keywords section, which is not visible to customers and is used solely for app discoverability, similar to searching in a journal database [22]. Discoverability on Google Play Store is based on what keywords developers include in the title, app description, and developer name [23]. We did not include any brand names in our search terminology as we did not want to bias our search strategy for specific apps. In addition, given that “vapes” and “e-cigarettes” are the only nonslang terms used to refer to this activity, and developers would not be exclusively using slang terms to refer to vaping as their key words, these terms were deemed sufficient to identify all vaping cessation apps.

Apps needed to be available in English and specifically mention offering vaping cessation support within their description to be included. Exclusion criteria included apps that promoted vaping usage or solely addressed smoking cessation. We did not download or explore the apps ourselves; all data used in this study came from the reviews posted to the included apps. Some apps were only available to Android or Apple users or were only found on the Canadian or US store, while others were found in both stores and in both countries. The app search was conducted from August 19 to 25, 2022.

### Data Analysis

Two research assistants (RAs), authors DR and RN, reviewed and analyzed all the reviews. One RA (DR) had previous coding experience and had taken the behavior change technique taxonomy v1 qualitative coding training course for a previous study [24]. DR mentored the other RA (RN) on qualitative coding throughout the analysis process. We used deductive content analysis, which is the process of coding data according to an existing categorization matrix, to organize and interpret the reviews [25]. The analysis framework used in this study was inspired by the framework used by Milward et al [21] in their study exploring alcohol cessation app reviews. We applied the same primary categories to organize the reviews, which included content, functionality, and aesthetics, and the same secondary categories, where reviews were further organized according to whether they included praise, criticism, or recommendations for the app. In an effort to prevent researcher bias that can arise when developing codes, the research team wanted to use predetermined codes with deductive content analysis [26]. The framework used by Milward et al [21] was appropriate for this analysis because it was developed to analyze app reviews for apps helping with addiction, with the only key difference between these studies being a focus on drinking habits versus vaping habits. Additionally, we adapted Milward et al’s [21] analysis framework by combining “general comments” and “other” into one larger “other” category that included general comments and specific comments that could not be categorized into the topics of content, functionality, or aesthetics. All 310 reviews gathered during data collection were included in the analysis process, and no reviews were discarded due to minimal information. Reviews providing simple, general comments with limited information for coding were included in the “other” category.

Using NVivo 14 software, 2 RAs (DR and RN) independently coded a sample of reviews using the adapted deductive analysis approach described earlier. Following this, both RAs met to confirm that this analysis approach was effective for analyzing the reviews. During this meeting, the RAs agreed that when analyzing the reviews, a fourth category had emerged from the data relating to the cost of apps. Prior to the inclusion of cost as a category, all specific comments that were unable to be categorized into content, functionality, or aesthetics were placed into the “other” category. Among the comments in “other,” the only pattern to appear were comments discussing cost; beyond this, there were no other trends or patterns, and no 2 comments addressed the same topic. With this information, the research team decided it was important to include comments addressing cost as a separate primary category. An inductive approach was then used to analyze data related to cost. As such, reviews were organized into 5 primary categories: content, functionality, aesthetics, cost, and other. Each primary category was further divided into 3 secondary categories: praise, criticism, and recommendations.

After the addition of cost as a primary category, the “other” category became dedicated to general comments (ie, “great app” or “helpful app”) and specific comments that could not be categorized under content, functionality, aesthetics, or cost. To prevent any overlap between categories during data analysis, specific definitions were developed for each primary category (see Table 1). Content referred to app features designed to support vaping cessation, such as time and e-cigarette use trackers, money counters, and online community forums. Functionality described how each app operates and included any technical problems that hindered users’ ability to engage with the app. Aesthetics was related to the design and visual appearance of the app. Cost included all reviews that mentioned having or not having a payment associated with the app.

**Table 1:** Primary categories and their associated definitions used for deductive content analysis of vaping cessation app user reviews.

Primary category	Definition
Content	Refers to app features designed to support vaping cessation, such as time and e-cigarette^a^ use trackers, money counters, and online community forums
Functionality	Describes how the app operates, and includes any technical problems that hinder users’ ability to engage with the app
Aesthetics	Relates to the design and visual appearance of the app
Cost	Includes all reviews that mention having or not having a payment associated with the app
Other	Includes general comments (ie, “great app” or “helpful app”) and specific comments that cannot be categorized under content, functionality, aesthetics, or cost

^a^e-cigarette: electronic cigarette.

After the 5 primary categories were finalized, both RAs then independently analyzed all remaining reviews using this analysis approach. Once the RAs had coded all the reviews, they met to discuss any discrepancies. Next, RN further subdivided reviews within each secondary category; if 2 or more of the references voiced the same opinion, they were grouped under a tertiary category. In an effort to make the analysis concise and identify emerging patterns in the data, ideas voiced in only one reference were grouped into an “only mentioned once” tertiary category that could be found under most secondary categories. RN reviewed the organization of the data into tertiary categories with the research team and resolved any discrepancies.

Reviews often included multiple ideas that spoke to different categories; hence, only the relevant section of each review was placed under the appropriate primary, secondary, or tertiary category, with each unique section of a review making up one reference. As a result, one review often resulted in multiple references. Therefore, the total number of references used in this study was larger than the total number of reviews. References were placed directly under the appropriate secondary category, which is why there were no numbers associated with the primary categories.

## Results

### App Details

In total, 17 apps supporting vaping cessation were found, of which 11 (64.7%) had user reviews. All vaping cessation apps with user reviews were included in our study, for a total of 11 apps and 310 reviews (see Table 2). Of the 11 apps, 9 (81.8%) were specific to vaping cessation, while 2 (18.2%) offered support for both vaping and smoking cessation. Of the 11 apps included in this study, 10 (90.9%) were first released between 2019 and 2022. It is unclear whether the 2 (18.2%) apps addressing smoking and vaping cessation always provided vaping cessation support or whether the vaping cessation component was added in a later version to what was once a smoking cessation app exclusively.

There were 310 reviews relating to 11 vaping cessation apps. As mentioned in the *Methods* section, there were more references than there were reviews, since one review could generate multiple references. The results were aligned with the primary categories the reviews were grouped into during data analysis. As such, the reviews were divided into 5 primary categories (content, functionality, aesthetics, cost, and other) and 3 secondary categories (praise, criticism, and recommendations).

**Table 2:** English-language vaping cessation apps with user reviews from the Canadian or US Apple App Store or Android Google Play Store as of August 2022.

App name	Year of release	Reviews (N=310), n (%)
Aeris: Quit Smoking & Vaping	2020	86 (27.7)
Brave the Crave	2019	1 (0.3)
Easy Quit Smoking and Vaping	Apple: 2009 Android: 2011	99 (31.9)
Escape the Vape	2022	25 (8.1)
No Vape – Crush Cravings	2019	22 (7.1)
Puff Count	2019	20 (6.5)
Quash – Quit Vaping	2021	3 (1.0)
Quit Vaping	2020	20 (6.5)
Quit Vaping Addiction Calendar	2020	10 (3.2)
Quit Vaping – For Good	2019	13 (4.2)
Quuit – Quit Vaping Now	2020	11 (3.5)

### Content

Content was the category with the highest number of references in this study, the majority of which were positive. In total, 103 references praised the content, 13 criticized the content, and 17 contained recommendations relating to content (Table 3).

**Table 3:** Results from deductive content analysis of vaping cessation app reviews including the primary category “content,” its secondary and tertiary categories, and their associated reference frequencies.

Secondary and tertiary categories			References, n (%)
**Praise (n=103 references)**			
	Hypnosis audio sessions	29 (28.2)	
	Money saved counter	20 (19.4)	
	Health information	15 (14.6)	
	Vape free timer	15 (14.6)	
	Vape use tracker	14 (13.6)	
	Social support	13 (12.6)	
	Free quit line	5 (4.9)	
	Personal advice	2 (1.9)	
	Triggers diary	2 (1.9)	
	Quit plan	2 (1.9)	
	Games	2 (1.9)	
	Nonspecific	13 (12.6)	
	Content praise mentioned once	12 (11.7)	
**Criticism (n=13 references)**			
	Tracker limitations	7 (53.8)	
	Content criticism mentioned once	6 (46.2)	
**Recommendations (n=17 references)**			
	Trackers	6 (35.3)	
	Social support	3 (17.6)	
	Widget	3 (17.6)	
	Content recommendation mentioned once	6 (35.3)	

#### Praise

Of the 103 references praising the app content and features, many comments simply stated that they “liked” the feature or found it “helpful” without specifying how. These were placed in the *nonspecific* tertiary subcategory (n=13, 12.6%). Overall, there were 11 different features that were mentioned and praised in more than 1 review, each making up a tertiary category. Here, we provide an overview of the most frequently praised features.

The *hypnosis audio sessions* tertiary category (n=29, 28.2%) was associated with the highest number of references. However, this feature was only available in 2 (18.2%) of the 11 apps, Quuit – Quit Vaping Now and Easy Quit Smoking & Vaping, which are centered around using hypnosis to quit vaping. The reviews were long and positive, usually mentioning being doubtful at first of the credibility of hypnosis as a cessation tool. However, multiple reviewers reported a noticeable change within the first few days. For example, 1 (3.4%) review stated:

I was not even that determined to quit!!! Listened to it once only and although the cravings were there they felt very distant. Feels very easy...

Trackers or counters were found in most vaping cessation apps and were frequently praised in reviews. The *money saved counter* (n=20, 19.4%) helped users realize the financial impact of vaping. For example, 1 (5%) reference stated:

I never realized how much money I spent on pods until this app laid it all out for me. I’m living paycheck to paycheck not realizing that by cutting out vaping I’d save so much money!

The *vape free timer* (n=15, 14.6%) offered users motivation and encouragement to stay vape free. One reference described how the user would experience “a dopamine spike better than what nicotine can offer” upon seeing how many days they had been vape free and how much money they had saved since quitting. An additional 2 (13.3%) references compared the experience of using trackers on the app to playing a game, explaining how “it almost gives you the feeling of a game’s high score that you’re trying to beat yourself.” In comparison to the *vape free timer*, reviews revealed that the *vape use tracker* (n=14, 13.6%) can help those who still actively vape, regardless of their intention to quit, to track their consumption and get a clearer picture of their vaping habits:

Puff Count brought to my attention how much I was vaping, which ultimately helped me decrease the amount I was vaping.

#### Criticism

The number of negative comments criticizing the content was drastically lower than the number of references praising the content. In total, 13 references criticized the content. Criticism mostly reflected what was lacking from the app, such as it “doesn’t give you motivational updates,” or obvious oversights, such as the news section being “stuck in 2019.” The only tertiary category that emerged was *tracker limitations* (n=7, 53.8%). Reviewers highlighted limitations on what they could track (ie, app tracking cigarette intake rather than vaping puffs), how they could track (ie, no ability to delete false entries), and when they could track (ie, quit day cannot be prior to the day the app was downloaded).

#### Recommendations

There were 17 references that featured recommendations relating to the content and features of the app. Similar to content criticism, the tertiary category with the highest number of references was related to improving the user’s experience with *trackers* (n=6, 35.3%). Reviewers wished they could track their vape use in more detail (ie, input the specific nicotine level of each puff), wished for more flexibility (ie, being able to delete and re-enter false entries and edit previous entries), and specified different ways they would like the collected data to be presented (ie, “a metrics chart where you can look at the week or the month” to view overall progress rather than viewing 1 day at a time). Reviewers also wanted a *widget* (n=3, 17.6%) that would allow them to input vaping use into a tracker without having to open the app. Additionally, others suggested ways *social support* (n=3, 17.6%) aspects could be incorporated into the app, such as a “chat box” to share “coping mechanisms” with other users.

### Functionality

Functionality had the second highest number of references, with the majority of references in this category being negative. There were 5 references praising functionality, 58 criticizing functionality, and 8 describing recommendations to improve the app (Table 4). When discussing functionality users tended to focus on whether the app operated as intended or not. However, some users described their experiences accessing technical support. Reviewers left short, general comments in the praise section and described in more detail the technical issues they encountered in the criticism section.

**Table 4:** Results from deductive content analysis of vaping cessation app reviews including the primary category “functionality,” its secondary and tertiary categories, and their associated reference frequencies.

Secondary and tertiary categories			References, n (%)
**Praise (n=5 references)**			
	Operating as intended	3 (60.0)	
	Technical support	2 (40.0)	
**Criticism (n=58 references)**			
	Whole app unusable	29 (50.0)	
	Specific features unusable	28 (48.3)	
	Functionality criticism mentioned once	1 (1.7)	
**Recommendations (n=8 references)**			
	Functionality recommendation mentioned once	8 (100.0)	

#### Praise

Of the 5 references praising the functionality of the apps, 3 (60%) praised the apps for *operating as intended*; 2 (66.7%) of them stated that the apps worked smoothly, while another explained how the recent updates streamlined downloading. An additional 2 (40%) references spoke positively about the *technical support* available on the apps. For example, 1 (50%) reference stated:

If an app isn’t acting right, chances are there’s a conflict that isn’t the [application’s] fault. Contact the dev – he’s very responsive.

#### Criticism

Complaints concerning the functionality of the apps were featured in 58 references. Users encountered technical issues at various points while using the apps, starting from the sign-in/log-in process to issues with specific features on the apps, such as audio files not downloading or inactive in-app links. Some issues rendered the *whole app unusable* (n=29, 50%), while others rendered a *specific feature unusable* (n=28, 48.3%). Regarding issues in the *whole app unusable* category, reviewers described glitches that prevented them from gaining further access to the apps or using any other feature of the apps, for example:

I can’t even open it to use it. It just gets stuck on the “looking for resources to download” screen and nothing ever happens and I can’t get any further into the app.

In the *specific feature unusable* category, glitching only impacted one feature. Reviewers described a variety of problems, such as trackers miscalculating or restarting count, chats being deleted, games not working, in-app links leading to an “Error” page, and more. One reference explained how malfunctions with one feature can render an app useless despite being able to navigate the rest of the app, for example, a hypnosis app where the audio sessions do not work.

#### Recommendations

Eight references provided recommendations relating to the functionality of the apps. No patterns emerged as no suggestion was repeated in more than 1 reference. Some requests were general, such as requesting more “updates” and “bug fixes” and asking the developers to “read [their] error logs.” Other suggestions were more specific, such as making the Apple version align consistently with the Android version and wanting to receive “haptic feedback upon pressing ‘log’ so [users are] not left wondering if [they] pressed it while it loads.”

### Aesthetics

Overall, this category had the fewest references. There were 8 references praising the aesthetics of the apps, specifically the *design* (n=4, 50%) and the *layout* (n=4, 50%) of the apps. For example, 1 (12.5%) reference stated, “It’s beautifully designed.” No criticism or recommendations specific to aesthetics were made (Table 5).

**Table 5:** Results from deductive content analysis of vaping cessation app reviews including the primary category “aesthetics,” its tertiary categories, and their associated reference frequencies.

Tertiary category under the secondary category “praise”	References (n=100), n (%)
Design	4 (50.0)
Layout	4 (50.0)

### Cost

Reviewers had varied opinions regarding the cost of an app, with 27 references praising cost, 38 references criticizing cost, and 1 reference providing a recommendation (Table 6). Comments discussing cost arose from both free apps and apps that required an upfront payment or heavily relied on in-app purchases. It is important to note that not all comments criticizing aspects related to cost were directed toward apps requiring payment; similarly, not all comments praising cost were directed toward free apps.

**Table 6:** Results from deductive content analysis of vaping cessation app reviews including the primary category “cost,” its secondary and tertiary categories, and their associated reference frequencies.

Secondary and tertiary categories			References, n (%)
**Praise (n=27 references)**			
	Satisfied with purchase	17 (63.0)	
	Support free of charge	7 (25.9)	
	Recommends premium version	3 (11.1)	
**Criticism (n=38 references)**			
	Not satisfied with purchase	15 (39.5)	
	Not satisfied with free version	12 (31.6)	
	Not transparent about cost	4 (10.5)	
	Costs money	4 (10.5)	
	Cost criticism mentioned once	3 (7.9)	
**Recommendations (reference=1)**			
	Cost recommendation mentioned once	1 (100.0)	

#### Praise

There were 27 positive references related to cost. Some reviewers were *satisfied with their purchase* (n=17, 63%), and others went ahead and *recommended the premium version* (n=3, 11.1%) to other users in order to access additional features. Some reviewers believed it was worth the purchase since it freed them from a deadly habit:

After all the money I’ve spent trying to quit smoking, I can’t believe it only cost me 5 bucks to actually do it!

Other users highlighted how purchasing the premium version was “pretty cheap compared to buying a pack of cigarettes,” making it a financially wise decision.

The *content that was free of charge* category (n=7, 25.9%), be it a free app or the free version of an app with in-app purchases, was also praised by many reviewers. Two references suggested that the cost of an app reflected the values of its developers, and thus, a free app implied that the developers are “genuine” and aim to help others first before establishing a profitable business. For example:

You can tell they really care about the people they want to help, and they obviously aren’t doing it for the money. No ads, no in-app purchases. Just folks wanting to help you get healthier!

#### Criticism

There were 38 negative references related to cost. Some users who had paid for the app or in-app purchases were *unsatisfied with their purchase* (n=15, 39.5%). These users expressed increased frustration when there was a technical issue with the app, some of whom demanded to be reimbursed. Others were *unsatisfied with the free version* (n=12, 31.6%) of an app that included in-app purchases. The free version was often described as “very bare-bones.” Some references simply stated that the *app costs money* (n=4, 10.5%), however, there was a clear undertone of frustration, for example, “Monthly subscription? Come on.” Furthermore, 3 (7.9%) references criticized the *lack of transparency* related to in-app purchases, for example:

I downloaded this because it said all of the content was free […] The first thing it did was ask me to sign up for a paid subscription, or a 7 day trial. Bye.

Three references stated that charging a fee for an app intended to help quit an addiction reflected greed:

Greedy […] It’s ridiculous they want us to quit then exploit us for money because we’re desperate. There are people out there wanting to quit and most young teens (who are the ones who should be quitting) and they don’t have the extra money to be able to use this app to try and [quit] nicotine.

#### Recommendations

There was only 1 reference that included a recommendation relating to cost. It asked for more transparency when it comes to in-app purchases:

Need to add to the description that this is a subscription-based app.

### Other

Most reviews in this category were general, not specifying which aspect of the apps they were speaking to, while some reviews spoke of a specific aspect in the user’s experience that did not fit within the topics of content, functionality, aesthetics, or cost. There were 95 (91.3%) references that praised the apps vaguely without providing much detail as to why they were beneficial. Reviewers often described how an app was “helpful” and “easy to use,” sharing success stories of how they successfully cut down their use of e-cigarettes or quit vaping altogether. In contrast, 8 (7.7%) references criticized a specific aspect of the apps that did not fit within content, functionality, aesthetics, or cost, such as, “can’t connect to [G]oogle,” “takes too long to install,” or “not consistent with the corresponding android application.”

Complaints were random, no patterns emerged, and no complaint was voiced by more than 1 reference. One reference provided a recommendation, suggesting that developers reduce the age limit to allow younger users the ability to access vaping cessation support, as well as create a sister app for cannabis cessation (Table 7).

These results provided an immense amount of data into the insights of users, with some distinctly notable findings. The majority of users found the content on the apps helpful, with the most popular features including *hypnosis audio sessions* (n=29, 28.2%), a *money saved counter* (n=20, 19.4%), *health information* (n=15, 14.6%) about how vaping impacts the body, a *vape free timer* (n=15, 14.6%) to track quit time, and a *vape use tracker* (n=14, 13.6%) for users still vaping. There were 17 recommendations on how current features could be enhanced or suggested features that could be added. Nevertheless, there were still 13 criticisms pertaining to app content, including disappointment over the absence of specific features and dislike over components of tracking features. Most reviews regarding the topic of functionality were negative, discussing technical issues that made either a *specific feature unusable* (n=28, 48.3%), such as audio files not playing, or the *whole app unusable* (n=29, 50%), such as the app not opening. On the topic of cost, most references praising cost included users of paid apps being *satisfied with their purchase* (n=17, 63%) and, alternatively, users of free apps (including those containing in-app purchases) being grateful for what was available for free (n=7, 25.9%). Conversely, many references criticizing cost included users of paid apps being unsatisfied with their purchase (n=15, 39.5%) and users of the free version of apps being disappointed with what the free version provides (n=12, 31.6%). No patterns emerged within the findings from the primary categories “aesthetics” and “other.”

**Table 7:** Results from deductive content analysis of vaping cessation app reviews including the primary category “other,” its secondary categories, and their associated reference frequencies.

Secondary category	References (n=104), n (%)
Praise	95 (91.3)
Criticism	8 (7.7)
Recommendations	1 (1.0)

## Discussion

### Principal Findings

This study focused on the experiences of individuals using mobile vaping cessation apps from app reviews on Apple App Store and Android Google Play Store. To date, there are no studies analyzing user reviews of apps developed to support vaping cessation. Our study results address an apparent gap in the knowledge on vaping cessation interventions, particularly user evaluations of available vaping cessation apps as mHealth interventions.

Individuals who use vaping cessation apps appear to be either most concerned with or most inclined to comment on aspects of apps that relate to content since the greatest number of reviews in this study referred to content. The majority of the comments regarding content were written in a positive tone, with users praising various features available on the apps. Comments criticizing content were minimal and included limitations of app features, strategies to improve those features, and features that had not been maintained and were outdated.

The first noteworthy feature, incorporated into 75% of vaping cessation apps, was trackers [8]. Tracking features in the apps were given particularly positive reviews. Based on reviews, apps contain trackers to evaluate various components of the quitting journey, including monetary benefits, days since the last vape puff, and vaping consumption in a given time frame for current vapers inquiring about their vaping frequency. Trackers were likely mentioned frequently in reviews because users found this feature exceptionally helpful. Our findings suggest that users find trackers helpful for their cessation journey as trackers offer motivation to follow through with their goal. Our results align with findings from studies on smoking cessation apps, which found that users appreciate tracking features and find them helpful for monitoring their progress and smoking frequency [18,27]. Studies have also found that users desire additional tracking features, including tracking health improvements from cessation duration, nicotine avoided, and total electronic juice (e-juice) avoided based on prior vaping habits [8,18]. These previous findings align with a review from our study suggesting the inclusion of a tracker for the amount of nicotine users are inhaling per puff. Overall, findings from this study are consistent with previous research suggesting that tracking features are a promising area for developers to expand on. Multiple users also provided suggestions on ways to further optimize the benefits tracking features can have. For example, users drew attention to the value in the display of tracker data, such as through a monthly metrics chart. Furthermore, users from our study recommended the addition of a phone widget for tracking so they can record their vape puffs without having to open the app. Studies of smoking app reviews have highlighted similar app improvements, such as the importance of a visual display of tracker data [18] and a widget to overcome the barrier of remembering to input when they smoke on an app in real time [18,27].

The second feature that users raved about in reviews was hypnosis soundtracks. Guided-hypnosis sessions aim to put people in a trance, allowing them to concentrate on weakening their urges to smoke/vape and increase their willpower to quit [28]. Users were so appreciative of these soundtracks that many thanked the developer in their reviews. Users emphasized how surprised they were that this technique was so effective for cessation. However, although this feature received solely positive reviews, reviews came from only 2 (18.2%) of the 11 apps. As such, it is unknown whether this feature is available in other apps and not used or not included in other apps. It is important to acknowledge that of the 2 (18.2%) hypnosis apps, 1 (50%) was specifically vaping focused (Quuit – Quit Vaping Now), while 1 (50%) was designed to address both vaping and smoking cessation (Easy Quit Smoking & Vaping). The app specific to vaping contained a total of 11 reviews, of which 10 were positive, and 3 of these positive reviews specifically praised the hypnosis feature. Given these positive findings, the exploration of hypnosis practices for vaping cessation may hold promising hope for e-cigarette users as new strategies for cessation are tested.

When commenting on the functionality of an app, reviews were typically critical. Issues noted with app functionality included those that prevented users from using specific features on the app and those that prevented users from using the app overall. Issues with the functionality of apps likely resulted in criticism because they hindered and, in some cases, completely inhibited the user’s ability to explore using these apps to help quit vaping. Various issues were noted with the functionality of app features, but it is important to note this included both trackers and hypnosis soundtracks. Users commented on tracker issues involving glitching and difficulty inputting data, as well as issues with hypnosis audio soundtracks not playing. Considering both these features were frequently mentioned in reviews as being helpful for quitting, improperly functioning features may impose a barrier to cessation for individuals using these apps. Similarly, concerns with trackers malfunctioning and apps glitching and crashing were identified in a study of smoking cessation app reviews [18]. Issues concerning technological difficulties have been connected to decreased retention of those using apps [29,30]. Our study identified that users of a hypnosis app show gratitude toward the developer that helped them resolve their issues when they contacted the developer directly. Contacting the developer directly may feel more personable and provide users with more reassurance than relying on reviews to gain the attention of the developer. To support app retention and prevent attrition, developers should work with users to address functionality issues.

A pattern emerged when comparing functionality and content. Comments regarding app functionality were primarily critical, while comments about content primarily offered praise. This suggests that users are inclined to comment on negative functionality experiences when the app is not functioning as expected but perhaps unlikely to comment on positive functionality experiences because it is an expectation that an app should function as intended. In contrast, users may be less inclined to comment on negative experiences with content, as opposed to positive experiences with app content, because users can ignore features they are uninterested in using. This provides impetus for developers to use creative features that have not yet been explored when designing vaping cessation apps, since at best these features will be appreciated and praised by users and at worst not used and ignored.

The notion of cost, although not included in the original analysis framework designed by Milward et al [21], was commented on so frequently in reviews that it necessitated its own theme. This suggests that the cost of an app, or lack thereof, holds great importance for users. Cost is one of the main factors contributing to the usage of mHealth apps, with barriers to app usage including the cost of the app itself, as well as hidden costs apparent only after downloading the app [31,32]. In this study, multiple users commented on the fact that it seemed contradictory for developers, who designed an app to help others improve their health through vaping cessation, to be capitalizing on the needs of individuals requiring support for cessation. In this same manner, it was thought that developers of free vaping cessation apps and those allowing advertisements to be removed for free are more genuine. Furthermore, users were frustrated when they downloaded a free app only to discover that most features were only accessible with the paid version of the app. Our findings align with a review of qualitative studies on mHealth apps that found that barriers to app usage include costs associated with full access to app features, free versions offering limited features, and having to pay to remove advertisements [33].

It is important for developers to consider what limitations they may be imposing for users when apps are not free and whether this counteracts their intention behind designing an app. Developers should consider how they can financially maintain the app, while limiting or mitigating the cost for users. If the functioning of an app is contingent upon a cost, then developers should consider what cost is appropriate, given what the app has to offer. Furthermore, developers should recognize they are potentially implementing a barrier when requiring payment for an app or an upgrade to the full version, which may limit access to vaping cessation support for some individuals. This emphasizes the importance for developers to be transparent with the cost of an app and which features are available with the free version compared to the premium version.

### Strengths and Implications

This is the first study to analyze the experiences of users of vaping cessation apps via user reviews. There are remarkably more apps developed for smoking cessation than vaping cessation. The low quantity of vaping cessation apps available on app stores today indicates a gap in current vaping interventions and identifies this as an optimal area for developers to pursue. As evident through the positive experiences many users shared, vaping cessation apps are valuable for individuals seeking vaping cessation, which provides a motive for developers to update apps to include user recommendations and resolve issues experienced by users. Findings from this study can provide direction to developers creating new cessation apps and provide guidance when making improvements to enhance the value and use of current apps. Developers should ensure app features account for a variety of contexts in which individuals may have chosen to use a vaping cessation app, allow users to personalize features to tailor them to their cessation context, and are updated frequently. Users have provided a variety of suggestions developers could use to improve tracking features, including adding a tracker of the amount of nicotine users are inhaling per puff, altering the display of tracker data to show weekly and monthly trends, and including a widget for easy tracking of vaping puffs. Additionally, developers could consider incorporating aspects of hypnosis into their cessation apps as users reported this feature to be particularly helpful, second only to tracking and counting features. In terms of the functionality of cessation apps, developers need to ensure they are efficiently addressing any ongoing problems. To support this, developers should provide contact information for users to report problems and regularly monitor user reviews.

As previously outlined, reviews from vaping cessation apps show many similar findings to reviews from smoking cessation apps. Indeed, although similarities exist between the experiences of smoking cessation and vaping cessation, there are distinct barriers that exist for e-cigarette users. Barriers unique to vaping cessation include the enjoyment of flavors, the discreteness of devices, and a lack of stigma [34]. Developers should consider this when developing apps for vaping cessation as reviews demonstrate that current vaping cessation app features tend to mirror those previously developed for smoking cessation apps. In line with this, developers should consider exploring innovative features developed specifically for vaping as our findings indicate that individuals are likely not disadvantaged by features they are not interested in using, and vaping-specific features have the potential to further enhance the benefits vaping cessation apps can have.

This study provides researchers of mHealth apps with a foundational layer of knowledge on the experiences of users of vaping cessation apps. However, some of our findings would benefit from further research to investigate why certain aspects of vaping cessation apps are helping users in the manner they suggest in reviews. More specifically, given the significant praise toward hypnosis features, further research is needed to explore the uptake and use of hypnosis amongst vapers and investigate how hypnosis uniquely supports vaping cessation. In addition, with the dynamic environment of app development, ongoing research into the efficacy of innovative features designed specifically for vaping will need to be conducted to ensure available cessation apps incorporate the most effective tools for cessation. Additionally, pertaining to the robust discussion around the cost of apps, it would be beneficial to further explore how financial barriers to vaping cessation support can be alleviated to ensure equity for individuals aspiring to quit. Most importantly, given the characteristics of app reviews, that they do not allow for further inquiry and do not provide ongoing information on app adherence and cessation, there is a need for both further qualitative research and quantitative research on this topic. Among smoking cessation apps, 31% of apps used in 2021 were no longer functioning in 2022 [35]. This highlights the need for a longitudinal quantitative study to provide information about app adherence, app availability, and abstinence rates. Interview-based qualitative studies will be able to gather comprehensive information to further explore the individual experience with vaping cessation apps and delve deeper into the reasoning behind app praise and criticism than what has been revealed in reviews. This will promote comparison between vaping and smoking cessation app user experiences and help identify what features and benefits may cross over.

### Limitations

There are a few limitations of our study that should be noted. First, we were not able to analyze all apps available for vaping cessation, because some apps did not have any reviews. However, it can be assumed that the number of reviews available increases somewhat proportionally to the number of users of the app. This implies that the most pertinent apps were likely analyzed by our study, as apps without reviews are likely used by fewer people or are newer additions to the app store. Second, 2 of the 11 apps included were designed to address both smoking and vaping cessation. For these 2 apps, it was difficult to differentiate which reviews referred to smoking and which to vaping, since some did not specify. Therefore, since all reviews were included, our findings contain some reviews discussing smoking cessation. However, this proved useful to identify possible differences in cessation, including identifying that 1 of the apps appears to be developed for dual cessation rather than individual smoking or vaping cessation. This suggests that apps can boost their viability by offering the option to select dual cessation. Third, reviews were often short and provided limited context. Given that researchers did not have the opportunity for follow-up as they do when conducting interviews, our analysis was confined to our interpretation of the reviews. Our interpretation may have differed from what the users had intended. However, the rigor applied in analysis mitigated this limitation by coding reviews with similar words and phrases into the same categories. Fourth, despite being hopeful that all available app reviews are authentic, we acknowledge that false reviews may be included in our study. Finally, since apps undergo regular updates, criticism provided in reviews may no longer be relevant if that aspect has already been fixed or updated. However, the feedback itself in the review is still important as it highlights what aspects are considered important, can impede app usage, and should be addressed first by developers. If this limitation exists, this proves that developers are responding to user concerns and recommendations.

### Conclusion

This study revealed the nature of app user reviews as it pertains to vaping cessation app content, functionality, aesthetics, and cost. A unique finding emerged relating to the successful use of hypnosis as a tool for vaping cessation. This finding is relevant for both developers, highlighting a feature to consider incorporating into more cessation apps, as well as for researchers to further explore the nuances of hypnosis and how it promotes cessation in the context of vaping. Furthermore, our study found that individuals express concerns regarding the intentions behind the development of cessation apps requiring payment. Users were apprehensive that they were being exploited for attempting to quit vaping. Developers must consider this finding seriously when developing apps and attempt to mitigate app payment, when possible. Understanding the experiences of those using apps can help developers better target and respond to the needs of those on their cessation journey and ensure app stores offer high-quality interventions that effectively support users in their vaping cessation journeys. Our study forms a basis that future studies can expand on and continue to support the development of interventions in this field.

## Abbreviations

e-cigarette: electronic cigarette
mHealth: mobile health
RA: research assistant

## Data Availability

The dataset analyzed in this study is available from the corresponding author upon request.
